# A novel t(3;13)(q13;q12) translocation fusing FLT3 with GOLGB1: toward myeloid/lymphoid neoplasms with eosinophilia and rearrangement of FLT3?

**DOI:** 10.1038/leu.2016.304

**Published:** 2016-12-02

**Authors:** E Troadec, S Dobbelstein, P Bertrand, N Faumont, F Trimoreau, M Touati, J Chauzeix, B Petit, D Bordessoule, J Feuillard, C Bastard, N Gachard

**Affiliations:** 1 Laboratory of Hematology, CBRS, CHU de Limoges et UMR CNRS 7276, Limoges, France; 2 Department of Oncology Genetics, Centre Henri Becquerel, Rue d'Amiens, Rouen, France; 3 Clinical Hematology and Cellular Therapy, CHU Dupuytren, Limoges, France; 4 Laboratory of Pathology, CHU Dupuytren, Limoges, France

According to the 2016 World Health Organization classification, myeloid neoplasms with eosinophilia (MPN-Eo) are associated with genetic abnormalities of genes coding for type III tyrosine kinase (TK) receptors, mainly PDGFRA, PDGFRB and FGFR1, but also JAK2.^1^ Beside these translocations, very rare FLT3 gene rearrangements have been reported, which raises the double question of its association with myeloid neoplasms and of its specific targeted therapy.^2, 3, 4, 5, 6, 7^

A new t(3;13)(q13;q12) was found from a case of atypical mixed lymphoid/myeloid neoplasm. This case, diagnosed MPN-Eo, was characterized by the coexistence of bone marrow myeloproliferation with circulating hypereosinophilia and T-cell lymphoblastic lymphoma in lymph node (Supplementary Results for detailed description). The patient could not benefit from new tyrosine kinase inhibitors. Evolution was fatal in 3 months despite conventional CHOP chemotherapy (Cyclophosphamide, Hydroxydaunorubicin, Oncovin and Prednisolone).

Karyotype of tumor cells from lymph nodes and bone marrow revealed a single clonal t(3;13)(q13;q12) translocation (Figure 1a, left panel). Absence of *FGFR1* gene rearrangement was checked by fluorescence *in situ* hybridization (FISH) and RT-PCR according to methods described by others.^8^ BCR-ABL gene translocation, FLT3-ITD and D835 mutation were also absent. FISH walking on both chromosomes 3 and 13 with BAC and fosmid probes showed that the breakpoint was located in a 58.6 kb region encompassing *HCLS1* and *GOLGB1* on chromosome 3 and in a 65.5 kb region containing the *FLT3* locus on chromosome 13 (Figure 1a, right panel).

*FLT3* maps to band q12 of chromosome 13 and *GOLGB1* to chromosome band 3q13. We hypothesized that this translocation would lead to a fusion transcript. Since the breakpoint region covered 15 out of the 23 exons of the *GOLGB1* gene, we hypothesized that *GOLGB1* gene could be a fusion partner. *FLT3* gene was the only candidate on chromosome 13. A multiplex PCR amplified a specific product located between exons 13 and 15 of *GOLGB1* and *FLT3* respectively (Figure 1b). Direct sequencing showed that this 2000 bp PCR product was specific. The rearrangement fused exons 14 of both *GOLGB1* and *FLT3* genes. Moreover, 36 bp of intron 14 of *GOLGB1* were inserted between the two exons 14 of *GOLGB1* and *FLT3* (Figure 1c). The genomic fragment corresponding to the der(3) contains the 5′sequence of *GOLGB1* fused in frame to the 3′ sequence of *FLT3* at nucleotide 8841 which corresponds to the beginning of exon 14. Genomic DNA sequencing showed that breakpoints were within *GOLGB1* intron 14 and *FLT3* exon 14 (not shown).

This t(3;13)(q13;q12) translocation identifies GOLGB1 as a new partner of FLT3. GOLGB1 encodes for giantin, a golgin subfamily B member 1 and the largest golgi complex-associated protein (372 kD), with numerous coiled-coil regions. GOLGB1-FLT3 protein fused together the three coiled-coil GOLGB1 domains with the split kinase TK domain of FLT3, that could lead to a constitutively multimerized active protein. Alternatively, constitutive TK activation could be due to the loss of the inhibitory juxtamembrane domain of FLT3, as reported for FIP1L1-PDGFRα gene rearrangement.^9^ GOLGB1 has been recently reported as a fusion partner with PDGFRB in a t(3;5)(q13;q33) translocation in a male patient with MLN-Eos.^10^ PDGFRB has also been reported to be fused with another golgin subfamily member, GOLGA4.^11^ The other published FLT3 partners, ETV6 helix-loop-helix and SPTBN1 coiled-coil domains exhibit spontaneous multimerization, at least theoretically for SPTBN1.^4^ ETV6–FLT3 fusion protein is indeed oligomerized and is constitutively activated.^12^ This emphasizes that, like SPTBN1 and ETV6, golgin family proteins participate in oncogenesis due to their ability to multimerize the TK partner domain.

To date, seven other cases of myeloid neoplasms with *FLT3* gene rearrangement have been published (Table 1). Five had an ETV6/FLT3 rearrangement, three of them with MPN-Eo associated with T-cell lymphoma, either peripheral or lymphoblastic, and two with a chronic MPN-Eo.^2, 3, 5, 7^ One case has been diagnosed as an atypical chronic myeloid leukemia, but with eosinophilia, and corresponded to a t(2;13;2;21)(p13;q12;q33;q11.2) with SPTBN1/FLT3 gene rearrangement.^4^ The last case was diagnosed as an atypical MPN with a B ALL and systemic mastocytosis corresponding to a t(13;13)(q12;q22) for which the FLT3 partner has not been identified.^6^

In the t(3;13)(q13;q12) translocation described here, the *FLT3* breakpoint was located within intron 14 just upstream from the exons coding for the TK domain. *FLT3* breakpoints in other published translocations are all located between exon 13 and 15 (Table 1). We also noticed that the *FLT3* exon 14 breakpoint was located within an Alu I restriction site and was embedded between non-coding Alu sequences, AluSz and AluJb (Figure 1d). On the other side, the breakpoint in *GOLGB1* was also found between two Alu sequences, AluSc and AluS. This led us to look for Alu repeats in the other published partners of FLT3 gene rearrangement, *ETV6* and *SPTBN1*.^4^ AluS, AluY sequences were located close to the 5′ end of the ETV6 breakpoint meanwhile AluYa1 sequence was located close to the corresponding 3′ end. For STPBN1, the breakpoint was not precisely located but was reported to be within the intron 14, which is 3 kb long and contains one AluSz sequence. Altogether, this highlights a nonrandom distribution of chromosome breakpoints both for FLT3 and its partners. Alu rich genomic region are known to render the genome sensitive to double-strand DNA breaks, which suggests the existence of a breakpoint cluster region. FLT3 gene rearrangement could thus involve reactivation of ancestral retrotransposon Alu sequences, as reported for BCR and ABL1, resulting in a looped-out chromatin conformation during interphase with double DNA strand breaks.^13^

The entire 10 kb *GOLGB1-FLT3* cDNA encodes a theoretical protein of 377 kDa. This full length mRNA was retrotranscribed from the lymph node tumor of the patient and the cDNA was cloned into the pcDNA3 eukaryote expression vector. No additional mutations were found after sequencing the whole *GOLGB1-FLT3* cDNA. Stable transfection of the *GOLGB1-FLT3* pcDNA3 vector was successful in the 32D myeloblastic cell line but failed despite repetitive attempts in the BA/F3 lymphoblastic cell line. Both cell lines were grown in an IL-3-dependent manner. The stably transfected 32D clone, named cl.2, expressed a protein of ~350 kDa (Figure 1e right panel). Immunoprecipitation of this protein with an antibody against the N terminal moiety of GOLGB1 was revealed with an antibody against the C-terminal moiety of FLT3 as well as with the 4G10 antibody raised against phosphorylated tyrosine motives (Figure 1e middle and left panel, respectively). Cl.2 cells expressing the GOLGB1-FLT3 fusion protein grew in the absence of IL-3 in contrast to clones expressing vector only and the parental 32D cell line, which died rapidly (Figure 1f). Constitutive phosphorylation of Erk and Akt was increased in these cells in the absence of IL-3 (Figure 1g).

To test if GOLGB1-FLT3 transfected cells were sensitive to targeted TK inhibition, transformed clones were cultured in the presence of four TK inhibitors also known to block FLT3: Imatinib, Midostaurine, Sorafenib and Ponatinib. Transformed cell growth was inhibited in a dose-dependent fashion by Midostaurine, Sorafenib and Ponatinib, but not by Imatinib (Supplementary Figure 1). The GI50s were 3682 nM for Imatinib (vector control, 826.2 nM), 0.85 nM for Ponatinib (vector control 501.5 nM), 0.65 nM for PKC412 (vector control, 23.22 nM). Sensitivity for Sorafenib was so high that GI50s could not be calculated precisely with the range of concentration used (vector control, 112.2 nM).

Therefore, our results on the functional characterization of the fusion protein argue in favor of direct transformation ability by constitutive FLT3 TK activity. Its transformation potential was also evidenced in a mouse model.^14^ Most cases, including ours, were diagnosed as MLN-Eos, with a rapid fatal issue in the absence of allogenic bone marrow transplantation. SPTBN1-FLT3–transformed Ba/F3 cells were sensitive to several FLT3 inhibitors.^4^ The therapeutic efficacy of FLT3 inhibitor has been described in patients with ETV6–FLT3 positive MLN-Eos.^2, 7^ In our case, the giantin-FLT3 transformed 32D cells were sensitive to different tyrosine kinase inhibitors, with in particular, a high specific activity for Sorafenib. Other new molecules such as those derived from ibrutinib have been reported as very potent and specific inhibitors of FLT3-ITD (Internal Tandem Duplication) product in FLT3-ITD positive acute myeloid leukemia.^15^ These new drugs could also be interesting in case of FLT3 gene rearrangement, as the target is the ATP pocket of FLT3 TK domain.

Altogether, including this new t(3;13)(q13;q12) translocation, MLN-Eos with FLT3 gene rearrangements exhibit close clinical features, similar genetic structures of their translocation with possible involvement of Alu sites, the same three-dimensional organization of the chimeric protein and high sensitivity (at least *in vitro*) to new TK inhibitor. This highlights the importance of FLT3 gene rearrangements at diagnosis and for adapted therapeutics rand raises the question a subgroup of MLN-Eos specifically associated with *FLT3* gene translocation, as suggested by some authors.^2^

## Figures and Tables

**Figure 1:**
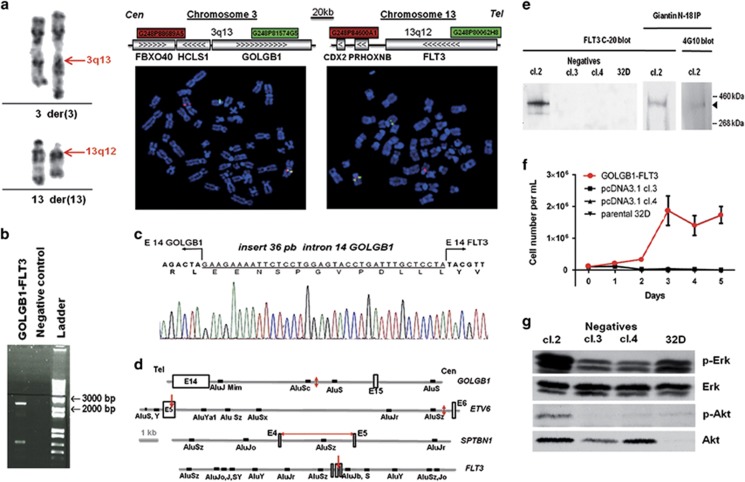
(**a**) Cytogenetic and FISH analysis of t(3;13)(q13;q12). Partial reverse heat giemsa-banded karyotype showing the t(3;13) translocation (left panel). With FISH analysis, split of der(3) and der(13) was clear with the two-color-labeled fosmid probes, G248P88689A5 (red signal), G248P81574G5 (green signal) for the 3q13 locus, and G248P84600A1 (red signal), G248P80062H8 (green signal) for the 13q12 locus, meanwhile the red and green signals were colocalized on the normal 3 and 13 chromosomes (right panel). (**b**) Identification of the *GOLGB1-*FLT3 fusion transcript. A multiplex RT-PCR was designed to detect 2000 bp product of the *GOLGB1-FLT3* chimeric mRNA in leukemic cells (Supplementary Table 1). (**c**) Sequencing of the junction. RT-PCR product revealed an in-frame fusion between *GOLGB1* exon 14 and *FLT3* exon 14 with an insertion of 36 pb sequence derived from intron 14 of *GOLGB1*. (**d**) Breakpoint regions of *FLT3* gene and other gene partners with localization of Alu sequences. Solid red arrows indicate breakpoints. Empty black boxes indicate exons. (**e**) Detection of the chimeric protein in the cl.2 clone isolated from 32D cells stably transfected with the PCDNA3 expression vector in which the *GOLGB1-FLT3* chimeric cDNA was inserted. A protein of about 400 kD was detected in cl.2 cells with the FLT3 C-20 antibody. Meanwhile no signal was detectable in control clones transfected (neg cl.3 and neg cl.4) with the empty pCDNA3 vector (left panel). Total cell lysate immunoprecipitation with Giantin N-18 antibody followed by revelation with the FLT3 C-20 antibody (middle panel) or with 4G10 antibody raised against phosphorylated tyrosine motive (right panel) confirms detection of GOLGB1-FLT3 chimeric protein. (**f**) GOLGB1-FLT3 chimeric protein rendered 32D cells IL-3 independent. 32D cells expressing the GOLGB1-FLT3 fusion transcript (red curve) or the empty vector (pcDNA3.1 cl.3 and 4) and parental 32D cells were deprived of IL-3 for 48 h and cultured in IL-3-free medium at 1 10^5^ cells/ml. Cell numbers were counted at the indicated time points. The graph depicts the average of three independent experiments. (**g**) GOLGB1-FLT3 chimeric protein exhibited a constitutive FLT3 downstream active signal. Western blot detection of phospho Erk (p-Erk) and total Erk proteins (upper panel) and phospho Akt and total Akt proteins (lower panel) in IL-3 deprived cells.

**Table 1:** Summary of published cases of FLT3 translocation-related neoplasm with our case: MLN-Eos, PTCL, T-LBL, aCML, aMPN and B ALL

*Number of cases*	*Sex*	*Age (years)*	*Diagnosis*	*Cytogenetics*	*RT-PCR*	*References*
Three cases	3M	29–60	MLN-Eos+PTCL or T-LBL	t(12;13)(p13;q12)	ETV6 exon 4 to 6–FLT3 exon 14	^3, 7^
Two cases	2F	68, 40	MLN-Eos	t(12;13)(p13;q12)	ETV6 exon 5–FLT3 exon 14 or 15	^2, 5^
One case	F	32	aCML	t(2;13;2;21)(p13;q12;q33;q11.2)	SPTBN1 exon 3–FLT3 exon 13	^4^
One case	M	46	aMPN+B ALL+systemic mastocytosis	t(13;13)(q12;q22)	Not done	^6^
Our case	F	71	MLN-Eos+T-LBL	t(3;13)(q13;q12)	GOLGB1 exon 14–FLT3 exon 14	This study

Abbreviations: aCML, atypical chronic myeloid leukemia; aMPN, atypical myeloproliferative neoplasm; B ALL, B-cell acute lymphoblastic leukemia; F, female; M, male; MLN-Eos, myeloid/lymphoid neoplasms with eosinophilia; PTCL, peripheral T-cell lymphoma; T-LBL, T-cell lymphoblastic lymphoma.
