# Supplementary figures and images for: A novel t(3;13)(q13;q12) translocation fusing FLT3 with GOLGB1: toward myeloid/lymphoid neoplasms with eosinophilia and rearrangement of FLT3?
Source: Leukemia. 2016 Dec 2;31(2):514–7. doi: 10.1038/leu.2016.304 (PMC5292680; doi:10.1038/leu.2016.304)

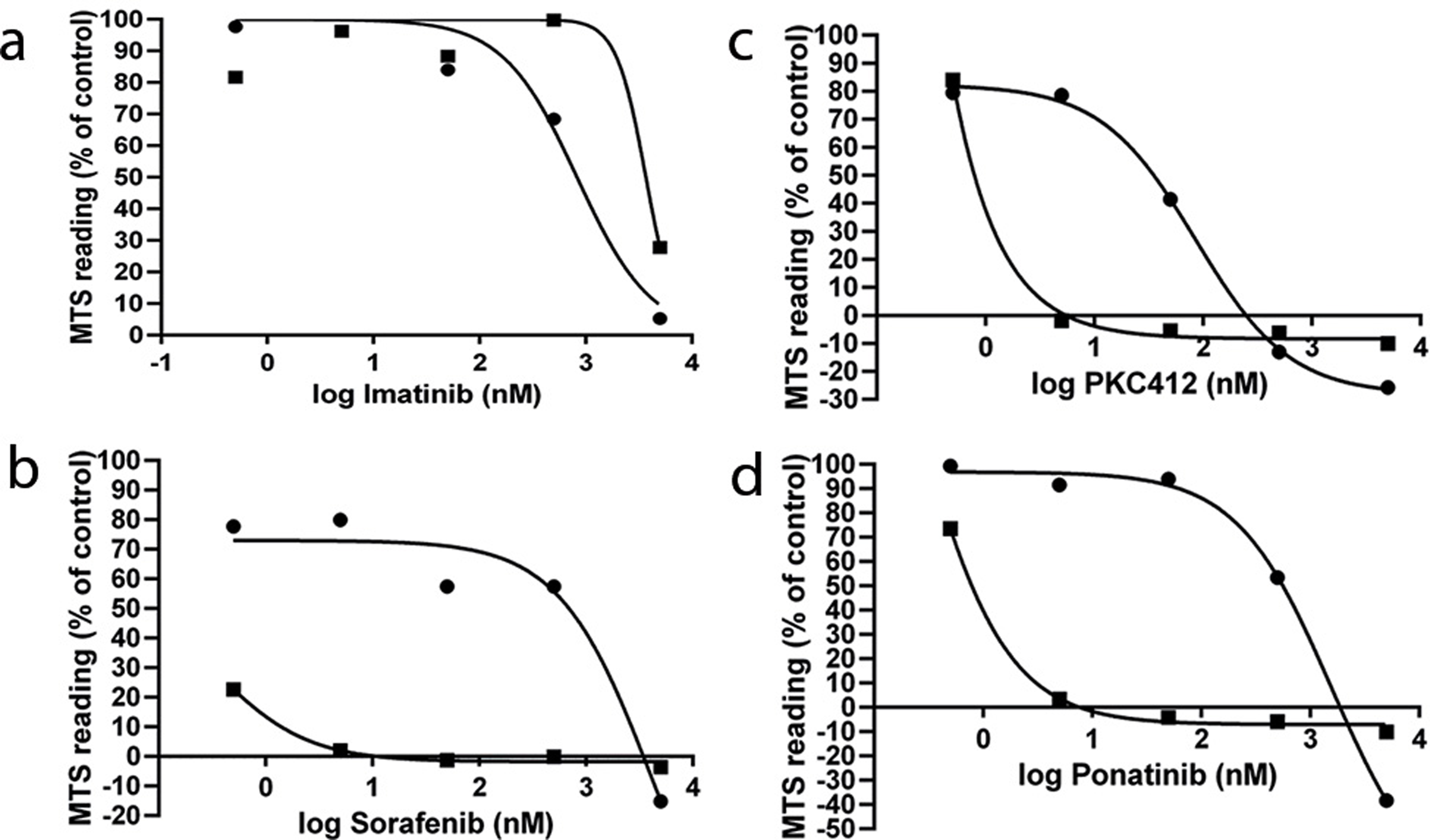

Supplement: Supplementary Figure 1 [file leu2016304x1.tif]

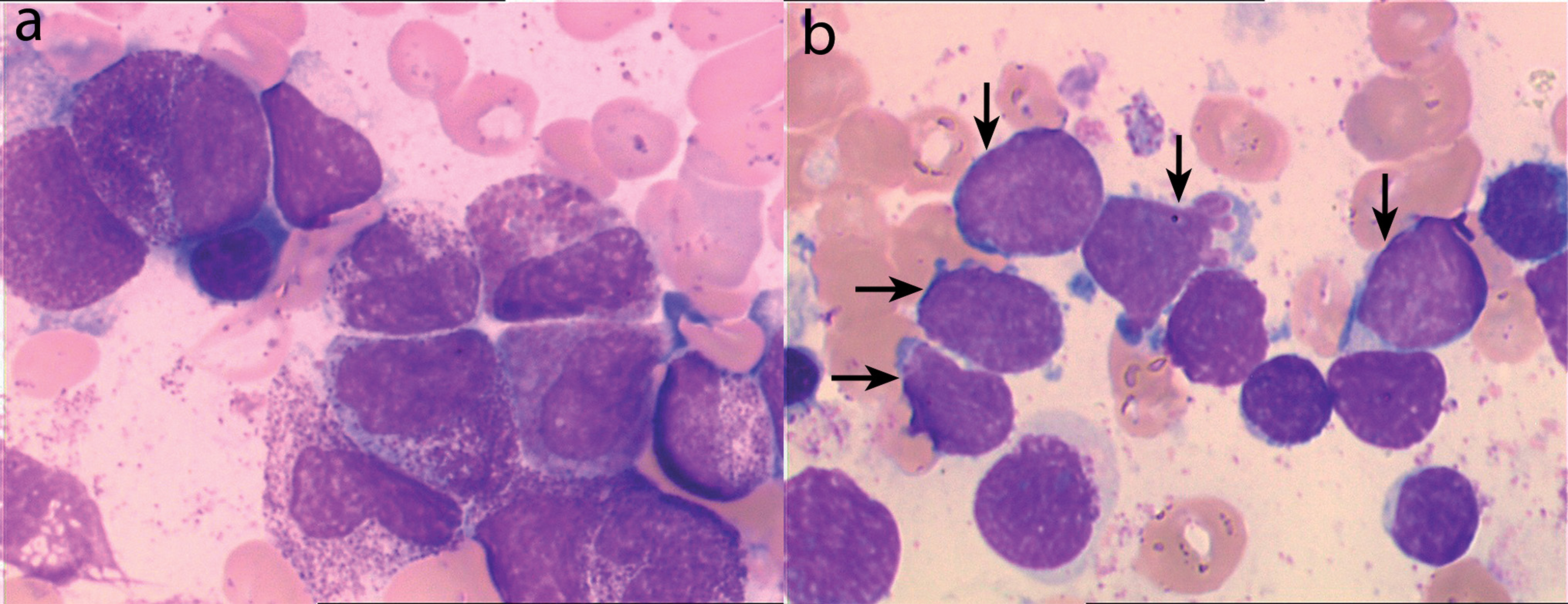

Supplement: Supplementary Figure 2 [file leu2016304x4.tif]
