# Supplementary material for: A novel t(3;13)(q13;q12) translocation fusing FLT3 with GOLGB1: toward myeloid/lymphoid neoplasms with eosinophilia and rearrangement of FLT3?
Source: Leukemia. 2016 Dec 2;31(2):514–7. doi: 10.1038/leu.2016.304 (PMC5292680; doi:10.1038/leu.2016.304)
Supplement: Supplementary Table 1 [file leu2016304x2.docx]

Supplementary table 1: Primers used in multiplex PCR for the detection GOLGB1-FLT3 fusion transcript

| GOLGB1-E2F | ATCAGGATTAGCAAATGTTGTTTTG |
| --- | --- |
| GOLGB1-E6F | GAATTTGTAATGATGAAGCAACAGC |
| GOLGB1-E10F | AACAACATGAAACAGCATCTCAGAC |
| GOLGB1-E13F | CAACTTCAGGAAAACTTGGACAGTA |
| FLT3-E23R | AAACGAAGTCAAATTAGGGAAGGAT |
| FLT3-E18R | ATGAAGCCCTGAGATTTGATCC |
| FLT3-E15R | GGATTGAGACTCCTGTTTTGCTAAT |
