# Supplementary material for: A novel t(3;13)(q13;q12) translocation fusing FLT3 with GOLGB1: toward myeloid/lymphoid neoplasms with eosinophilia and rearrangement of FLT3?
Source: Leukemia. 2016 Dec 2;31(2):514–7. doi: 10.1038/leu.2016.304 (PMC5292680; doi:10.1038/leu.2016.304)
Supplement: Supplementary Material [file leu2016304x3.docx]

**Supplementary Materials and Methods**

## Cytogenetics and fluorescence in situ hybridization (FISH)

RHG banded karyotype was performed as previously reported and described according to the International System for Human Cytogenetic Nomenclature.[^1-3^](#_ENREF_1) FISH techniques were performed as previously described.[^4^](#_ENREF_4) Briefly, FISH probes were prepared from bacterial artificial chromosome (BAC) clones and fosmids obtained from Bacpac Resources (CHORI, Oakland, CA, USA). The BAC and fosmids were located from chromosome 3q22.1 to 3q13.33 and covered chromosome 13q12 region. Probes were labeled with Spectrum Orange or Spectrum Green-dUTP using nick translation and were hybridized according to the manufacturer’s recommendations (Abbott Molecular, Rungis, France).

## Polymerase Chain Reaction and cloning

The GOLGB1-FLT3 fusion transcript was detected using reverse transcription–polymerase chain reaction (RT-PCR) from lymph node biopsy. For amplification, multiple forward primers were used in combination with several reverse primers (supplementary table 1). The set of the primers detecting the fusion transcript was E13F a GOLGB1-specific sense primer (5’ CAACTTCAGGAAAACTTGGACAGTA 3’) and E15R a FTL3-specific antisense primer (5’ GGATTGAGACTCCTGTTTTGCTAAT 3’).

Repeat content and position of Alu sequences were determined on 20Kb regions flanking breakpoints using the CENSOR website (<http://www.girinst.org/censor/index.php>).[^5-7^](#_ENREF_5)

The theoretical molecular weight of the protein was evaluated with SIB Bioinformatics Resource Portal (http://web.expasy.org/compute_pi). To clone the full length cDNA, reverse transcription of the full length fusion mRNA was first performed with the following primer: 5’-AGGGATGAAGTCCTTAAAACTAAATTG-3’. Then amplification of the 10 kb GOLGB1-FLT3 full length cDNA was done with the GOLGB1 upstream primer 5'-CTAGAAATGCTGAGCCGATTATCAG-3' and the FLT3 downstream primer 5’- GGTCGAAGATTCGTAGAGGAACAATTTA-3’ with the high fidelity polymerase from Agilent technologies (Les Ulis, France). Cloning of GOLGB1-FLT3 full length amplification products was performed in pCR-XL TOPO vector (Life Technologies, Carlsbad, CA, USA) and then in the pcDNA 3.1 expression vector (pCMV promoter). Sanger sequencing was performed as described.[^8^](#_ENREF_8)

## Cell culture, transfection and proliferation

The interleukin 3 (IL3) dependant 32D cell line was purchased from DSMZ (Brunswick, Germany). These cells were grown in RPMI 1640 culture medium plus 10% SVF and IL3 at 2 ng/ml (R & D systems, Minneapolis, USA). Before transfection, the pcDNA3 vector -Biolabs, Evry, France). Transfection and selection of cells was performed as described using 10 µg linearized vector for 10 million cells.[^9^](#_ENREF_9) Clones were selected for expression of the giantin-FLT3 fusion protein by western blot. For cell proliferation assays, cells were cultured in the absence of IL3. The number of viable cells was determined every 24h by trypan blue exclusion during 5 days.

## Western blot and immunoprecipitation

Whole protein extracts and immunoprecipitations were performed as described.[^10^](#_ENREF_10)

For Western blots, antibodies against FLT3/Flk2 (sc-479, SantaCruz Biotechnology, Heidelberg, Germany), Giantin N18 (sc-46993, SantaCruz Biotechnology), Akt (#99272, Cell Signaling Technology, Danvers, MA, USA), p-Akt1/2/3 (Ser 473)-R (sc-7985-R; SantaCruz Biotechnology), p44/42 MAPK (Erk1/2) antibody (#9102; Cell Signaling Technology), phospho-p44/42 MAPK (Erk1/2) (Thr202/Tyr204) antibody (#9101; Cell Signaling Technology) were used at 1/200, 1/200, 1/1000, 1/200, 1/1000 and 1/1000 respectively. The 4G10 antibody (Millipore, Molsheim, France) against phosphorylated tyrosines was used at 1/1000.

For immunoprecipitation, protein lysates (250 µg) were incubated 1h on ice with polyclonal antibody Giantin N18 (4 µg, sc-46993, SantaCruz Biotechnology), and 100 µl μMACS™ Protein A MicroBeads (Miltenyl Biotech).

## Tyrosine kinase inhibitor assays

Cells were seeded in triplicate at 2 10^4^ cells/100µL for each inhibitor concentration and controls and grown for 3 days. We tested four kinase inhibitors at different concentrations (0.5, 5, 50, 500 and 5000 nM): Ponatinib (sc-362710), Midostaurine (sc-200691.), Sorafenib (sc-280125) and Imatinib (sc-202180) (SantaCruz Biotechnology). Plates were analyzed after 72h and proliferation was measured by a colorimetric method using the Aqueous One Solution Cell Proliferation Assay kit (Promega, Southampton, UK). Graphs and 50% growth inhibitory (GI50) values were calculated using GraphPad PRISM 6 software (San Diego, CA, USA).

**Supplementary Results: detailed description of the case**

A 71-year-old woman was admitted in the University Hospital Centre of Limoges for asthenia and diffuse pruriginous rash in February 2005 without organomegaly. Leukocytes were at 45 G/L, with 6.8 G/L immature myeloid cells, 28 G/L mature neutrophils, 1 G/L eosinophils, 4.8 G/L monocytes, without blast cells. Hemoglobin was 111 g/L and platelet 185 G/L. LDH were normal. Bone marrow (BM) was characterized by a marked hypercellularity with 17% promyelocytes , 7% myelocytes, 9% metamyelocytes 8% immature eosinophils and 13% mature eosinophils, but without excess of blast and nor dysplasia. Conventional karyotype of BM cells was 46,XX,t(3;13)(q13;q12)[20]. Neither Bcr/Abl transcript nor FIP1L1-PDGFRα rearrangement could be detected by PCR.[^11^](#_ENREF_11)^,^ [^12^](#_ENREF_12) Based on chronic circulating leucocytosis with eosinophilia and circulating myeloid precursors and BM hypercellularity (supplementary figure 2a), a diagnosis of 8p11 syndrome was first suggested. Because of the presence of the non described clonal t(3;13)(q13;q12) chromosomal translocation, diagnosis of atypical myeloproliferative neoplasm was finally proposed. Initial treatment was symptomatic. In April 2005, the patient had lymph node, spleen and liver enlargement with general status deterioration. At this time, leucocytosis was 42 G/L with 27.6 G/L neutrophils, 5.1 G/L eosinophils, 2.9 G/L monocytes, 4 G/L myeloid precursors without circulating blast cells. LDH levels were increased at 874 UI/L**.** Cytology (supplementary figure 2b) and immunophenotypic analysis of lymph node biopsy revealed a T -cell lymphoblastic lymphoma, with expression of the CD2, CD5, CD7, CD1a markers and terminal deoxyribonucleotide transferase (TdT) marker (not shown). Expression of CD4, CD8, CD34 and surface CD3 was negative. Histopathological analysis of the BM biopsy confirmed both the diagnosis of MPN with myeloid hypecellularity and eosinophilia with concomitant T-cell lymphoblastic lymphoma (not shown). Despite CHOP treatment, the patient died 3 month after diagnosis.

## Supplementary References

1. Brigaudeau C, Trimoreau F, Gachard N, Rouzier E, Jaccard A, Bordessoule D*, et al.* Cytogenetic study of 30 patients with multiple myeloma: comparison of 3 and 6 day bone marrow cultures stimulated or not with cytokines by using a miniaturized karyotypic method. *Br J Haematol* 1997 Mar; **96**(3)**:** 594-600.

2. Sehested J. A simple method for R banding of human chromosomes, showing a pH-dependent connection between R and G bands. *Humangenetik* 1974 Jan 22; **21**(1)**:** 55-58.

3. Shaffer LG-J, JeanSchmid, M (Michael) International Standing Committee on Human Cytogenetic Nomenclature. *ISCN 2013 : an international system for human cytogenetic nomenclature (2013)*. Basel : Karger, c2013.

4. Bertrand P, Bastard C, Maingonnat C, Jardin F, Maisonneuve C, Courel MN*, et al.* Mapping of MYC breakpoints in 8q24 rearrangements involving non-immunoglobulin partners in B-cell lymphomas. *Leukemia* 2007 Mar; **21**(3)**:** 515-523.

5. Jurka J. Repeats in genomic DNA: mining and meaning. *Curr Opin Struct Biol* 1998 Jun; **8**(3)**:** 333-337.

6. Jurka J. Repbase update: a database and an electronic journal of repetitive elements. *Trends Genet* 2000 Sep; **16**(9)**:** 418-420.

7. Jurka J, Kapitonov VV, Pavlicek A, Klonowski P, Kohany O, Walichiewicz J. Repbase Update, a database of eukaryotic repetitive elements. *Cytogenet Genome Res* 2005; **110**(1-4)**:** 462-467.

8. Laforet MP, Turlure P, Lippert E, Cornillet-Lefebvre P, Pigneux A, Pradeau R*, et al.* Design and feasibility of a novel, rapid, and simple fluorescence 26-plex rt-PCR assay for simultaneous detection of 24 fusion transcripts in adult acute myeloid leukemia. *J Mol Diagn* 2013 Mar; **15**(2)**:** 186-195.

9. Asso-Bonnet M, Feuillard J, Ferreira V, Bissieres P, Tarantino N, Korner M*, et al.* Relationship between IkappaBalpha constitutive expression, TNFalpha synthesis, and apoptosis in EBV-infected lymphoblastoid cells. *Oncogene* 1998 Sep 24; **17**(12)**:** 1607-1615.

10. Chanut A, Duguet F, Marfak A, David A, Petit B, Parrens M*, et al.* RelA and RelB cross-talk and function in Epstein-Barr virus transformed B cells. *Leukemia* 2014 Apr; **28**(4)**:** 871-879.

11. Score J, Walz C, Jovanovic JV, Jones AV, Waghorn K, Hidalgo-Curtis C*, et al.* Detection and molecular monitoring of FIP1L1-PDGFRA-positive disease by analysis of patient-specific genomic DNA fusion junctions. *Leukemia* 2009 Feb; **23**(2)**:** 332-339.

12. van Dongen JJ, Macintyre EA, Gabert JA, Delabesse E, Rossi V, Saglio G*, et al.* Standardized RT-PCR analysis of fusion gene transcripts from chromosome aberrations in acute leukemia for detection of minimal residual disease. Report of the BIOMED-1 Concerted Action: investigation of minimal residual disease in acute leukemia. *Leukemia* 1999 Dec; **13**(12)**:** 1901-1928.
