# Supplementary material for: A novel t(3;13)(q13;q12) translocation fusing FLT3 with GOLGB1: toward myeloid/lymphoid neoplasms with eosinophilia and rearrangement of FLT3?
Source: Leukemia. 2016 Dec 2;31(2):514–7. doi: 10.1038/leu.2016.304 (PMC5292680; doi:10.1038/leu.2016.304)
Supplement: Supplementary Figure Legend [file leu2016304x5.docx]

**Supplementary Figure legends**

**Supplementary Figure 1. Dose-response curves to (a) Imatinib, (b) PKC412, (c) Sorafenib and (d) Ponatinib**. Plots indicate the growth response of an empty vector transfected clone grown in the presence of interleukin-3 (IL-3), squares indicate Giantin-Flt3 transformed clone grown in the absence of IL-3. On the y axis, results of proliferation are expressed as a ratio compared to cell proliferation in absence of the inhibitor (% of control).

**Supplementary Figure 2. Cytology**

A: Bone marrow smear at diagnosis: slides were May-Grünwald/Giemsa stained, revealing hypergranular immature granulocytes without maturation arrest and with high levels of mature neutrophils (x 1000).

B: Axillary lymph node imprint 2 month after: small undifferentiated blasts are pointed by the arrow (x 500).
